# Investigation of the Correlation Between Selected miRNAs, Proinflammatory Cytokines, and Serum Trace Elements in Bladder Cancer Development and Progression

**DOI:** 10.3390/cimb48010053

**Published:** 2025-12-31

**Authors:** Arzu Ay, Nevra Alkanli, Engin Atli, Hakan Gurkan, Pinar Koroglu, Hasan Can Kuvan, Muhidin Hassan Ibrahim, Gokhan Cevik, Necdet Sut

**Affiliations:** 1 Department of Biophysics, Faculty of Medicine, Trakya University, Edirne 22030, Türkiye; 2 Department of Biophysics, Faculty of Medicine, Haliç University, Istanbul 34060, Türkiye; 3 Department of Medical Genetics, Faculty of Medicine, Trakya University, Edirne 22030, Türkiye; 4 Department of Histology and Embryology, Faculty of Medicine, Haliç University, Istanbul 34060, Türkiye; 5 Department of Urology, Faculty of Medicine, Trakya University, Edirne 22030, Türkiye; 6 Department of Biostatistics and Medical Informatics, Faculty of Medicine, Trakya University, Edirne 22030, Türkiye

**Keywords:** bladder cancer, microRNA, proinflammatory cytokine, trace element, biomarker

## Abstract

In our study, we aimed to investigate the relationship between miRNA-21, miRNA-155, miRNA-34a, IL-6, TGF-β, and TNF-α expression levels and serum trace element levels in the development and progression of bladder cancer. RT-PCR was used to establish miRNA-21, miRNA-155, and miRNA-34a expression levels while serum IL-6, TGF-β, and TNF-α levels were determined using the ELISA and measured with an atomic absorption spectrophotometer. In the patient group, miRNA-21 and miRNA-155 expression levels were significantly higher compared with the healthy control group (*p* < 0.001). Furthermore, in the patient group, miRNA-34a expression was significantly lower compared with the control group (*p* < 0.001). IL-6, TNF-α, TGF-β, copper levels, and the copper to zinc ratio were significantly higher in the patient group (*p* < 0.001). Serum iron and zinc levels in the patient group were significantly lower compared with the control group (*p* < 0.001). There was a significant positive correlation between miRNA-155 and IL-6 and TNF-α (r = 0.279, *p* = 0.015*; r = 0.325**, *p* = 0.004). A significant positive correlation was detected between miRNA-34a and IL-6 and TGF-β (r = 0.294*, *p* = 0.010; r = 0.447**, *p* < 0.001). By evaluating these important biomarkers together, it might be possible to implement clinical applications for bladder cancer treatment and develop individual therapeutic strategies.

## 1. Introduction

Bladder cancer constitutes one of the ten most prevalent cancers worldwide, accounting for an estimated 430,000 newly diagnosed cases each year. Despite advances in therapy, bladder cancer poses a unique difficulty within the field of genitourinary malignancies due to its frequent recurrence and the highly variable prognosis observed among patients [1]. Bladder cancer is one of the most common types of cancer worldwide and is a significant cause of morbidity and mortality. Despite local treatments available to patients diagnosed with bladder cancer, there is a high risk of recurrence and variable progression of the disease. In some patients, poor clinical results can occur despite radical surgery or radiotherapy. Therefore, it is extremely important to elucidate the molecular mechanisms associated with early diagnosis and progression of bladder cancer [2,3]. Bladder cancer risk factors include chronic inflammation and genetic and environmental factors [4,5,6].

miRNAs play a role in regulating immune responses by modulating the expression of immunity-related genes. Cancer-related inflammation is associated with the production of inflammatory cytokines via extrinsic pathways associated with cancer-promoting conditions and intrinsic inflammatory pathways activated by genetic events [7,8].

In particular, miRNA-21 is upregulated in nearly all carcinomas and hematological malignancies. miRNA-21 is also induced in macrophages, blood mononuclear cells, and breast cells. miRNA-21 levels are reported to be associated with TGF-β induction and are significantly higher in transformed cells; studies have shown that recombinant TGF-β treatment induces miRNA-21 upregulation in bladder cancer cell lines. Therefore, miRNA-21 is considered a potential therapeutic target. Although TGF-β can function as a tumor suppressor in early tumorigenesis, it can also promote metastasis at more advanced stages. The TGF-β pathway can enhance or inhibit miRNA maturation [7,8].

miRNA-155 is considered the first miRNA that plays an oncogenic role. miRNA-155 is overexpressed in various cancers, such as leukemia, lymphoma, and colon, breast, lung, stomach, and pancreatic cancers. Additionally, miRNA-155 overexpression is associated with increased cytokine expression [7,8].

miRNA-34a is reported to be downregulated by TGF-β signaling. In cancer tissues overexpressing TGF-β, upregulated miRNAs can play a tumor-supporting role by downregulating the expression of tumor suppressor genes. Therefore, downregulation of miRNAs can be considered a strategy for cancer treatment. In various cancers, downregulation or inhibition of specific miRNAs can lead to oncogene overexpression and tumor-promoting effects. Increased expression of these miRNAs or downregulation of target genes is important for cancer treatment. A better understanding of the molecular and epigenetic mechanisms underlying the relationship between inflammatory cytokine signaling and miRNAs in cancer could enable the development of new therapeutic strategies targeting the tumor microenvironment [7,8].

Trace elements are environmental factors that facilitate the development of bladder cancer. Imbalances in trace elements, which are essential components of biological structures, are associated with the development of various cancer types [9,10,11]. Inflammatory conditions are associated with changes in systemic iron metabolism [12]. Copper is a trace element necessary for normal immune function [9,10,11]. Increases in ceruloplasmin levels following infection are associated with changes in copper concentrations. High concentrations of ceruloplasmin have been identified in disease states, with copper considered an antioxidant [12]. Zinc plays an anticarcinogenic role by stabilizing the structure of DNA, RNA, and ribosomes. Zinc is also effective in immune function. As a result of zinc deficiency, the production of some proinflammatory cytokines, such as IL-6, TGF-β, and TNF-α can increase [9]. Serum copper to zinc ratio is also important for evaluating the prognosis of patients with cancer in clinical practice. Since the inflammatory state can modulate copper and zinc homeostasis, the copper to zinc ratio is considered an important marker of inflammation [9,10].

Investigating the molecular pathways of bladder cancer is very important to identify potential markers that might be effective in the prognosis and progression of the disease. Detection of new genetic and environmental markers associated with inflammation in the etiopathogenesis of bladder cancer could be effective in establishing disease risk factors or predicting treatment efficacy and disease prognosis. A better understanding of the biological behavior of bladder tumors is important for the implementation of appropriate treatments with minimum morbidity and mortality and for the development of specific treatments against bladder tumors.

Therefore, in this study, we aimed to investigate the relationship between miRNA-21, miRNA-155, miRNA-34a, IL-6, TGF-β, and TNF-α proinflammatory cytokines, and serum iron, copper, and zinc levels with the early diagnosis and progression of bladder cancer.

## 2. Materials and Methods

### 2.1. Determining Patient and Control Groups and the Collection of Blood Samples

The study population consisted of individuals residing in the Thrace region of Türkiye, which constitutes the European area of the country. This region holds a unique position, acting historically as a major genetic and geographical corridor between Europe and Anatolia (near East). This population demonstrates an admixture pattern with substantial contributions from both southeastern European and Anatolian and near Eastern gene pools. Genetically, the population shows close affinity with neighboring Balkan nations (particularly Bulgaria and Greece) and Western Anatolian populations.

The study was conducted in accordance with the Declaration of Helsinki and was approved by the Ethics Committee of Trakya University Faculty of Medicine (protocol code TÜTF-GOBAEK 2020/262; approval date: 18 April 2022). Informed consent was obtained from all participants of the study. This study was supported by Trakya University Scientific Research Projects Unit (TUBAP) with project number 2022/176.

Blood samples were collected for routine examinations from both the patient group with bladder cancer and the control group. For our study, we used 2 mL of peripheral venous blood containing ethylenediaminetetraacetic acid (EDTA) per patient. The patient group included adults diagnosed with bladder cancer while the control group consisted of adults without bladder cancer. Patients and controls younger than age 19 years old, people diagnosed with blood diseases and coagulation disorders, people taking trace element supplements, and those diagnosed with any other malignancy were excluded from our study. We confirmed that all samples used for the atomic absorption spectrophotometer, ELISA, and miRNAs analyses were retrieved from −86 °C storage and used in a single, dedicated assay run. Therefore, all samples were subjected to no previous freeze–thaw cycles, which confirms the integrity of the collected biomolecules and trace elements.

### 2.2. RNA Isolation and Quality Control

Blood samples were collected into EDTA-containing tubes and total RNA was extracted immediately from whole blood using the InnuPrep RNA Mini Kit (Analytik Jena, Jena, Germany). The samples were processed immediately after collection. The time from venipuncture to the initiation of RNA isolation was recorded and averaged across all samples to ensure consistency. Hemolysis was assessed in all samples before analysis. We used a spectrophotometric method to measure free hemoglobin at 414 nm and samples showing evidence of considerable hemolysis were excluded from the analysis. Blood samples obtained from both patients with bladder cancer and control participants (collected for routine testing) were first centrifuged at 1000× *g* for 5 min and the resulting supernatant was discarded. The pellets were resuspended in 350 µL of lysis buffer. The mixtures were then transferred to a pre-cleaning column and centrifuged at maximum speed for 5 min. An equal volume of 70% ethanol was added to the resulting flow-through liquid. This final mixture was applied to the RNA binding column and centrifuged at maximum speed for 45 s. Subsequently, the column was washed sequentially by adding high and low stringency wash buffers (both containing alcohol). Following each wash buffer addition, the column was centrifuged at 12,000 rpm for 1 min. Lastly, to elute the RNA from the column, elution buffer was added and the total RNA was extracted following a final centrifugation at 8000 rpm for 1 min. The concentration and purity of the isolated total RNA were measured using the NanoDrop Spectrophotometer (Thermo Fisher Scientific, Wilmington, DE, USA), and RNA integrity was assessed using the Agilent 2100 Bioanalyzer (Agilent Technologies, Santa Clara, CA, USA) with only samples yielding an RNA integrity number greater than 7.0 being accepted for further analysis.

### 2.3. miRNA-21, miRNA-155, and miRNA-34a Expression Analysis

Real-time polymerase chain reactions (RT-PCR) using Taqman probes were adopted for measuring the expression levels of miRNA-21, miRNA-155, and miRNA-34a. The purified RNA samples were prepared with reagents, including Taqman PCR master mix and Taq Man Probe/Primer mixes, and run on a thermocycler at the optimized cycle temperatures. U6 RNA was used as the endogenous reference control; we did not use an exogenous spike-in control. U6, ΔCTE, ΔCTC, ΔΔCt, and 2^−ΔΔCt^ values were established for miRNA-21, miRNA-155, and miRNA-34a.

### 2.4. Determining Serum IL-6, TGF-β, and TNF-α Proinflammatory Cytokine Levels

Serum IL-6, TGF-β, and TNF-α proinflammatory cytokine levels were measured with the ELISA. Peripheral venous blood samples taken from patients and controls were centrifuged at 4000× *g* for 10 min and from which the serum samples were collected. These serum samples were stored at −86 °C until measurements were performed. Serum cytokine levels were determined using the ELISA according to the manufacturer’s protocol. For IL-6 (R&D Systems, Minneapolis, MN, USA; catalog no. D6050), sensitivity (or limit of detection) was 0.11 pg/mL, sample dilution factor was 1:2, and measurement type was standard. For TNF-α (Thermo Fisher Scientific, Waltham, MA, USA; catalog No. EHTNFA), sensitivity was 1.7 pg/mL, sample dilution factor was 1:1, and measurement type was standard. For TGF-β (Bio-Techne, Minneapolis, MN, USA; catalog no. DB100B), sensitivity was 3.0 pg/mL, sample dilution factor was 1:10, and measurement type was total (acid activated). Before measurement, serum samples were subjected to acid activation (incubation with 1 N HCl followed by neutralization with 1.2 N NaOH/0.5 M HEPES) to convert the latent form of TGF-β into its biologically active, measurable form. This step ensured that all circulating TGF-β was quantified. All serum trace elements (i.e., iron, copper, zinc) have been reported in mg/dL. The concentrations of iron, copper, and zinc in the diluted serum samples were measured using the atomic absorption spectrophotometer (Shimadzu AA-6800, Shimadzu Corporation, Kyoto, Japan), based on calibration curves.

### 2.5. Determining Serum Trace Element Levels

Blood samples were first processed by centrifugation at 5000 rpm for 5 min to separate the serum. For iron, copper, and zinc analysis, the isolates were diluted with distilled water to a final volume of 7 mL and thoroughly homogenized using a vortex mixer. Calibration graphs for iron, copper, and zinc concentrations were established using a series of standard solutions at concentrations of 0.5, 1.0, 1.5, 2.0, and 2.5 ppm (mg/L). The concentrations in the diluted serum samples were then measured using the atomic absorption spectrophotometer based on these calibration curves. The copper to zinc ratio was subsequently calculated for both patient and control groups. Linear calibration was confirmed across the biologically relevant range for serum, typically 50–250 g/dL for iron, copper, and zinc. Certified reference serum materials (e.g., Seronorm Trace Elements Serum or equivalent) were analyzed at two distinct concentration levels (low and high) with every batch of samples. All quality control measurements fell within ±2 standard deviations (SD) of the target values. Inter-assay coefficient of variation (CV) was consistently maintained below 7% across all measurement runs for iron, copper, and zinc. Intra-assay CV was maintained below 5% for all three elements. All serum samples were visually inspected immediately after separation. Any sample exhibiting macroscopic hemolysis (a clearly noticeable pink or red discoloration) was excluded from the study. We confirm that all separated serum and cellular fractions were immediately aliquoted into appropriate polypropylene tubes and stored continuously at −86 °C immediately following separation. This strict adherence to a single freeze–thaw cycle minimizes the risk of cellular lysis and protein degradation, thereby validating the stability and reliability of the measured trace element concentrations.

### 2.6. Statistical Analysis

Statistical analysis of the results was performed using IBM Statistics Package of Social Science version 20.0 software. For all continuous variables, the assumption of normal distribution was rigorously checked using Kolmogorov–Smirnov or Shapiro–Wilk tests before statistical analysis. Data exhibiting a normal distribution were presented as mean ± SD and analyzed using the *T*-test. Data that were not normally distributed was analyzed using the Mann–Whitney U test. By using the Mann–Whitney U test when *T*-test assumptions were not met, we avoided data transformation methods that could potentially lead to a loss of original data interpretability. The Chi-squared test was selected to examine whether the frequency distributions of categorical variables differed significantly between the two independent groups (i.e., patient versus control). The Chi-squared test is the most appropriate and widely accepted method for comparing categorical frequencies, making complex alternative methods unnecessary in this context.

Age, iron, copper, zinc, and copper to zinc ratio were compared between patient and control groups using the independent samples test. Diabetes, hypertension, cholesterol, heart disease, family history of cancer, family history of bladder cancer, smoking, and alcohol parameters were compared between both groups using the Chi-squared test. In addition, odds ratio risk estimate values were determined for these parameters. miRNA-21, miRNA-155, miRNA-34a, IL-6, TGF-β, and TNF-α values were compared between patient and control groups using Mann–Whitney U test. Additionally, correlation values were established for miRNA-21, miRNA-155, miRNA-34a, IL-6, TGF-β, TNF-α, iron, copper, zinc, and copper to zinc ratio parameters.

## 3. Results

The results of this study indicate that the group of patients diagnosed with bladder cancer had significant differences in specific risk factors and associated health conditions when compared with the control group. The mean age of the patient group was 66.15 ± 5.93 years, while the mean age of the control group was 64.00 ± 6.37 years. The mean ages were quite similar for both the patient and control groups, suggesting that the observed differences in other factors are less likely to be confounded by age and allow for a more focused interpretation of the reported associations. In the patient group, there were significantly more patients with hypertension were than in the control group (odds ratio [OR]: 7.405, 95% confidence interval [CI]: 3.376–16.244, *p* < 0.001). This finding means that patients with bladder cancer were approximately 7.4 times more likely to have hypertension than the control group, which indicates a very strong positive association between the two conditions. In the patient group, cholesterol levels were significantly higher than in the control group (OR: 5.508, 95% CI: 1.512–20.065, *p* = 0.010). Patients with bladder cancer were around 5.5 times more likely to have elevated cholesterol levels, suggesting a moderately strong association. In the patient group, the rate of heart disease was significantly higher than in the control group (OR: 11.294, 95% CI: 3.227–39.526, *p* < 0.001), which represents the strongest association found, indicating that patients with bladder cancer were approximately 11.3 times more likely to have a history of heart disease. This finding suggests potential common underlying risk factors or pathophysiological mechanisms linking cardiovascular health and bladder cancer. In the patient group, the frequency of patients with a family history of cancer was significantly higher than the control group (OR: 4.402, 95% CI: 2.079–9.321, *p* < 0.001). Patients with bladder cancer were around 4.4 times more likely to report a general family history of cancer, suggestive of a shared genetic predisposition that might increase susceptibility to various cancers, including bladder cancer. In the patient group, those with a family history of bladder cancer were significantly more frequent than in the control group (OR: 8.377, 95% CI: 1.832–38.306, *p* = 0.004). This is a strong risk factor, suggesting a strong influence of shared genetic factors or common environmental or lifestyle exposures within the family specific to this type of malignancy. The number of smokers in the patient group was significantly higher than in the control group (OR: 3.650, 95% CI: 1.845–7.219, *p* < 0.001). This observation aligns with established medical knowledge, as smoking is one of the most prominent and strong risk factors for bladder cancer. Carcinogens in tobacco smoke (particularly aromatic amines) are processed by the body and concentrated in the urine, damaging the lining of the bladder and causing cancerous changes (Table 1).

This study provides compelling evidence of a strong association between bladder cancer and several comorbidities and risk factors. The elevated rates of hypertension, high cholesterol, and heart disease suggest that cardiometabolic risk factors might share common biological pathways, such as chronic inflammation and oxidative stress with cancer development. High frequencies of both general and specific family cancer history underscore the importance of genetic susceptibility in identifying individuals at high risk. The confirmed strong association of bladder cancer with smoking highlights the crucial importance of smoking cessation as a primary prevention strategy for bladder cancer. These findings are valuable for screening and prevention efforts, suggesting that individuals with a history of hypertension, heart disease, or a family history of cancer should be considered for more vigilant monitoring regarding their bladder health.

The molecular data strongly suggests that the expression profiles of three specific microRNAs are significantly altered in patients with bladder cancer. These alterations point to their potential roles as biomarkers or even active drivers in the pathogenesis or development of the disease.

The mean miRNA-21 expression level was 16.40 ± 4.76 in the patient group and 1.90 ± 1.08 in the control group. In the patient group, the miRNA-21 expression level was significantly higher compared with the control group (*p* < 0.001). For miRNA-21, the striking difference in mean expression (over eight times higher in patients) suggests that it could be an excellent diagnostic biomarker for bladder cancer, and a potential therapeutic target.

While the mean miRNA-155 expression level was 4.06 ± 6.19 in the patient group, it was 1.13 ± 1.05 in the control group. In the bladder cancer patient group, the miRNA-155 expression level was significantly higher compared with the control group (*p* < 0.001), with this significant overexpression suggesting that it contributes to the oncogenic process in the bladder.

The mean miRNA-34a expression level was 1.01 ± 0.90 in the patient group and 1.54 ± 0.42 in the control group, which was a significant difference (*p* < 0.001). This significant downregulation or reduction in miRNA-34a expression removes the brake on cancer cell growth, allowing for uncontrolled proliferation and survival, which is a common characteristic observed in many cancers (Table 2).

These findings collectively support the idea that the dysregulation of these specific microRNAs is a major factor in bladder carcinogenesis (cancer development) and can offer excellent avenues for non-invasive diagnosis (e.g., urine or blood samples) and the development of miRNA-targeted therapies (e.g., anti-miRNA oligonucleotides to inhibit miRNA-21 or miRNA-155, or miRNA mimics to restore miRNA-34a).

The findings reveal a consistent pattern of upregulation for all three measured cytokines in patients with bladder cancer, which strongly points to a state of chronic inflammation and altered growth factor signaling that is characteristic of and potentially drives tumor progression.

The mean IL-6 expression level was 46.37 ± 18.12 in the patient group diagnosed and 18.58 ± 14.92 in the control group, which was a significant difference (*p* < 0.001). The mean IL-6 level in patients was nearly 2.5 times higher than in controls, indicating a robust inflammatory response associated with the presence of bladder cancer, which makes IL-6 a strong candidate as both a prognostic marker and a therapeutic target.

While the mean TNF-α expression level was 134.81 ± 39.31 in the patient group, it was 99.99 ± 21.88 in the control group; therefore, a significantly higher TNF-α expression level in the patient group compared with the control group (*p* < 0.001). It is thought that TNF-α is another crucial proinflammatory cytokine that often acts as a tumor promoter when chronically elevated.

**Figure 1:**
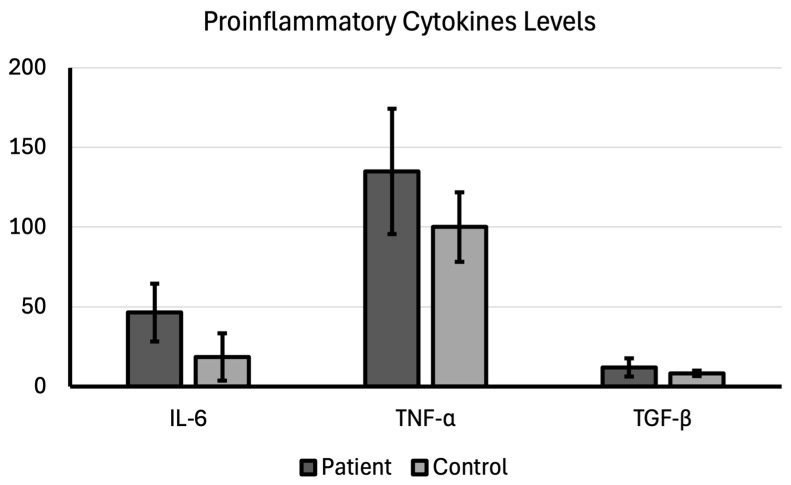
Proinflammatory cytokine levels compared between patient and control groups.

The mean TGF-β expression level was 12.07 ± 5.70 in the patient group diagnosed and 8.19 ± 1.68 in the control group, which was a significant difference (*p* < 0.001). It is thought that TGF-β is a pleiotropic (multiple-effect) cytokine with a complex dual role in cancer (Table 2; Figure 1).

These findings provide strong evidence that chronic, pro-tumorigenic signaling is a hallmark of bladder cancer. For IL-6 and TNF-α, the simultaneous, significant elevation of these two proinflammatory cytokines implies that bladder cancer exists within an environment of persistent inflammation. This environment facilitates genetic instability and provides survival signals to the malignant cells. The high level of TGF-β suggests that these tumors are likely leveraging this factor to promote invasiveness, suppress immune surveillance, and remodel the local tissue, indicating a potentially more aggressive phenotype.

This data provides compelling evidence regarding the considerable alteration of crucial trace elements—specifically iron, copper, and zinc—and their ratios in the blood serum of patients with bladder cancer compared with healthy controls. The findings reveal a consistent pattern of disturbed homeostasis (balance) of essential metal ions in the patient group. This dysregulation is closely linked to oxidative stress, inflammation, and altered enzyme activity, all of which are key features of cancer biology.

The mean serum iron level was 96.39 ± 6.61 in the patient group and 129.84 ± 5.19 in the control group, thus being significantly lower in the patient group (*p* < 0.001). Cancer and the associated chronic inflammation lead to increased production of hepcidin, a hormone that restricts iron release from storage cells and iron absorption in the gut. It is thought that this process results in functional iron deficiency in the serum. Rapidly proliferating cancer cells have a high demand for iron to support DNA synthesis and cell division.

This increased cellular uptake can contribute to lower serum levels. The mean serum copper level was 167.41 ± 17.33 in the patient group and 132.98 ± 6.30 in the control group. Serum copper level was significantly higher in the patient group compared with the control group (*p* < 0.001). Elevated copper levels are a consistent finding across many malignancies, driven by several pro-cancer mechanisms, such as angiogenesis, inflammation, oxidative stress, and cell proliferation.

While the mean serum zinc level was 99.33 ± 13.44 in the patient group, it was 124.70 ± 5.73 in the control group; which was a significant difference (*p* < 0.001). Low zinc levels can impair immune surveillance, allowing cancer cells to evade detection. It is thought that copper deficiency compromises the body’s ability to neutralize reactive oxygen species, exacerbating the oxidative stress caused by the high copper and inflammatory status.

**Figure 2:**
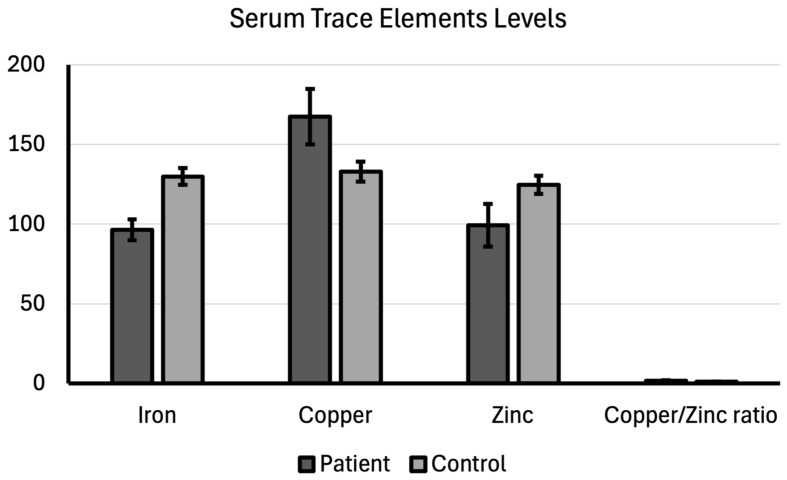
Serum trace elements levels compared between patient and control groups.

The average copper to zinc ratio was 1.71 ± 0.29 in the patient group and 1.07 ± 0.07 in the control group. The copper to zinc ratio was significantly higher in the patient group compared to the control group (*p* < 0.001; Table 2). The increased ratio reflects the dual changes—high copper and low zinc—and acts as a marker for the imbalance in redox (oxidation–reduction) state and inflammation. A high ratio indicates a pro-oxidant environment that is favorable for tumor growth and progression. These findings suggest that trace element balance is deeply integrated into the pathophysiology of bladder cancer, providing potential targets for nutritional intervention or novel metalloenzyme-based therapies (Figure 2).

This data provides a crucial network of correlations between the aforementioned molecular factors (e.g., miRNAs and cytokines) and trace element imbalances. These correlations suggest interconnected regulatory pathways that drive bladder cancer progression. The correlational analysis moves beyond simple association to suggest a complex regulatory network at play in patients with bladder cancer.

In the patient group, a significant positive correlation was observed between miRNA-21 and miRNA-155 (r = 0.232*, *p* = 0.046) and miRNA-34a (r = 0.382**, *p* = 0.001). A significant positive correlation was seen between miRNA-155 and IL-6 (r = 0.279, *p* = 0.015*) and TNF-α (r = 0.325**, *p* = 0.004). The strong positive correlation between miRNA-155 and both IL-6 and TNF-α positions miRNA-155 as a key link between molecular deregulation and chronic inflammation. There was a significant positive correlation between miRNA-34a and IL-6 (r = 0.294*, *p* = 0.010) and TGF-β (r = 0.447**, *p* < 0.001). Also, there was a significant positive correlation between IL-6 and TGF-β (r = 0.356**, *p* = 0.002). The simultaneous elevation and correlation between IL-6 and TGF-β highlights a coordinated system where inflammation directly facilitates the pro-metastatic and immunosuppressive environment. There was a significant negative correlation between TGF-β and the copper to zinc ratio (r = −0.235*, *p* = 0.042). Also, there was a significant positive correlation between serum copper levels and copper to zinc ratio (r = 0.675**, *p* = 0.000). However, there was a significant negative correlation between serum zinc levels and copper to zinc ratio (r = −0.669**, *p* = 0.000). The highly elevated copper to zinc ratio is firmly established as a consequence of both copper overload and zinc depletion, reflecting a highly pro-oxidant state that underlies the biological aggression signaled by the cytokine and miRNA data (Table 3).

In essence, the study reveals that the pathology of bladder cancer in these patients is driven by a feedback loop involving oncogenic miRNAs activating inflammation IL-6 or TNF-α, which coordinates with growth factors (e.g., TGF-β) and contributes to a severely dysregulated metal profile and a copper to zinc imbalance.

## 4. Discussion

Upregulated or downregulated miRNAs are reportedly associated with immune tolerance, potential autoimmunity, cancer initiation, and cancer progression. Inflammatory mediators known as cytokines could play an important role in regulating miRNA expression. Thus, cytokines might also contribute to the regulation of genes associated with inflammation and oncogenesis while miRNAs could regulate genes associated with the secretion of different cytokines [13,14].

miRNA-21 has oncogenic and onco-suppressive functions. Downregulated miRNA-21 expression is associated with impaired tumor progression, and miRNA-21 inhibition is considered a therapeutic approach in cancer treatment. In addition, since miRNA-21 can be transferred between cells via exosomes, it can be used as an important biomarker in the diagnosis and prognosis of cancer [15,16]. In a previous study, miRNA-21 was shown to induce TNF-α expression in patients with oral cancer, enhancing tumor progression [17]. It is thought that miRNA-21 might serve as a biomarker and an important regulator of molecular pathways in bladder cancer [15].

In our study, miRNA-21 expression was significantly higher in the patient group compared with the control group. The high expression levels of miRNA-21 in patients diagnosed with bladder cancer might be an important biomarker in the diagnosis and prognosis of patients with cancer, as this miRNA can be transferred between cells via exosomes. Therefore, miRNA-21 inhibition could be a therapeutic target in the treatment of bladder cancer [7].

miRNA-155 is considered another miRNA that plays an important role in cancer and inflammation. miRNA-155 is associated with leukemia and lymphoma, and breast, colon, lung, stomach, and pancreatic cancers. miRNA-155 plays an important role in targeting oncogenic suppressors or anti-inflammatory signaling pathways by promoting the progression of inflammatory pathologies [7]. In a previous study, overexpressed miRNA-155 levels were detected in urine samples of patients with non-muscle invasive bladder cancer [18,19]. Furthermore, miRNA-155 upregulation in bladder cancer is associated with tumorigenesis, in vitro proliferation, and increased in vivo tumorigenesis [20].

miRNA-155 expression was significantly higher in the patient group compared with the control group. In this study, significantly higher miRNA-155 expression levels in the patient group corroborates the results of a limited number of published studies. Significantly increased miRNA-155 expression levels could be a genetic biomarker promoting bladder cancer tumorigenesis; therefore, miRNA-155 inhibition might be effective in the treatment of bladder cancer [7].

miRNA-34a is an important member of the miRNA-34 family and is encoded by its own transcript. Ectopic re-expression of miRNA-34a in tumor-derived cell lines is associated with cell growth inhibition and altered miRNA-34 expression plays an important role in the pathogenesis of many cancers [7].

miRNA-34a expression levels were significantly lower in the patient group compared with the control group. miRNA-34a, which regulates the cell cycle and chemosensitivity of bladder cancer cells, can be considered as an important marker for tumor treatment. The downregulation of miRNA-34a in patients with bladder cancer could be an important genetic biomarker. As such, it is anticipated that miRNA-34a regulation in blood might be a prognostic and therapeutic marker for bladder cancer [13,21].

IL-6 is a pleiotropic inflammatory cytokine and is effective in regulating immune functions. IL-6 plays an important role in cancer-related inflammation and cancer progression processes. Significantly higher serum IL-6 levels were detected in patients with cancer inflammation [13,22].

Significantly higher IL-6 levels were seen in the patient group compared with controls. The overproduction of this protein cascade in patients with bladder cancer might be related to the inflammatory state. It is anticipated that by regulating the imbalance of IL-6 overproduction, there could be better treatment of bladder cancer-associated inflammation, thus IL-6 might serve as an important therapeutic molecule for bladder cancer [13,22].

TGF-β is a multifunctional cytokine and plays an important role in promoting or inhibiting tumor formation by regulating carcinoma initiation, metastasis, and progression. TGF-β is a cytokine that is effective in cancer-associated inflammation [13,23]. TGF-β is also important for regulating tumor progression [7].

There were significantly higher serum TGF-β expression levels in the patient group compared with the control group. It is anticipated that regulation of TGF-β overexpression imbalance could be important in treating bladder cancer progression due to the pleiotropic effects of this cytokine in the tumor microenvironment [13].

TNF-α is a cytokine and acts as an inflammatory mediator and is secreted by various cell types. Depending on the cell types, TNF-α can trigger immune cell activation by interacting with its receptors. This cellular activity is very important in tumor immune management. TNF-α can exhibit both pro-tumoral and anti-tumoral effects [13,24].

Serum TNF-α expression levels were significantly higher in the patient group compared with the control group. Due to the functional roles of TNF-α in the proliferation, activation, and differentiation of cancer and immune cells, regulation of TNF-α expression levels might be an important strategic approach for the development of effective treatments against bladder cancer.

Trace elements are also considered essential cofactors that play an important role in cancer development or inhibition [25]. Analysis of body fluids for trace elements is important for identifying potential biomarkers of early diagnosis, prognosis, and progression of various types of cancer, such as bladder cancer [10]. In studies conducted with different populations, serum copper levels and copper to zinc ratios are significantly higher in solid organ cancers, such as breast, ovarian, lung, cervical, prostate, and stomach cancers [25,26,27,28,29]. Serum copper levels in patients with bladder cancer were significantly higher than in healthy controls.

Zinc facilitates the structural stabilization of DNA, RNA, and ribosomes, and plays an anticarcinogenic role. However, zinc homeostasis can vary depending on the type of tissue. Therefore, the relationship between serum zinc levels and cancer progression is complex. Zinc can improve immune function by reducing oxidative stress, thus having a protective effect at the onset of cancer [25]. Increased zinc levels have been detected in some specific breast cancer tumors, melanoma, and liver cancer [30,31]. However, significantly lower serum zinc levels were found in other cancer types, such as ovarian, cervical, kidney, and bladder cancers [32,33,34,35,36]. In our study, serum zinc levels in patients were significantly lower compared with healthy controls.

Iron can increase the risk of cancer by contributing to the formation of free radicals; however, some studies have shown that iron can play an important role in preventing tumor development. Some studies have also reported that iron can increase the risk of tumors [36,37]. Serum iron levels in patients were significantly lower compared with healthy controls.

In addition, the copper to zinc ratio is an important marker for evaluating cancer progression. In a previous study, a significant increase in copper levels and a significant decrease in zinc levels (thus an increased copper to zinc ratio) were associated with testicular cancer induction [25]. The serum copper to zinc ratio was significantly higher in patients with bladder cancer compared with healthy controls.

Some miRNAs, such as miRNA-21, miRNA-155, and miRNA-34a play important roles in posttranscriptional gene regulation and are effective in controlling the inflammation cascade during cancer progression. Inflammatory mediators, such as IL-6, TGF-β, and TNF-α contribute to cancer-related inflammation via the interaction between inflammatory cells and tumor cells. These inflammatory mediators contribute to the inflammatory microenvironment, leading to an increased risk of cancer development. Thus, these mediators have functional roles in the proliferation, activation, and differentiation of cancer and immune cells [13]. Overexpression of inflammatory cytokines, such as IL-6, TGF-β, and TNF-α can trigger or inhibit cancer development. Various miRNAs, including miRNA-21, miRNA-155, and miRNA-34a are associated with cancer development and inflammation and can be controlled by inflammatory mediators [7]. When miRNA and cytokine expression levels were evaluated together in this study, significant positive correlations were seen between miRNA-21, miRNA-155, and miRNA-34a. Significant positive correlation was seen between IL-6 and TGF-β, IL-6 and miRNA-155 and miRNA-34a, TNF-α and miRNA-155, and TGF-β and miRNA-34a. Zinc deficiency is associated with increased production of proinflammatory cytokines, such as IL-6, TGF-β, and TNF-α. Additionally, imbalances in trace element levels of copper, zinc, and iron have been linked to the development of various types of inflammation-related cancers, such as bladder cancer [9,10].

When serum trace element levels and cytokine expression levels were evaluated together, a significant positive correlation was found between serum copper levels and the copper to zinc ratio, while a significant negative correlation was found between serum zinc levels and copper to zinc ratio. Additionally, a significant negative correlation was found between copper to zinc ratio and serum TGF-β expression levels.

In our study, it is possible that IL-6, TNF-α, and TGF-β cytokines might be effective in regulating miRNA-155 and miRNA-34a expression levels in the patient group and could also play a role in miRNA-21 expression, which shows a positive significant correlation with miRNA-155 and miRNA-34a expression levels. In addition, the negative significant correlation of the copper to zinc ratio with serum TGF-β expression levels suggests that TGF-β might also be effective in regulating the copper to zinc ratio. It might be possible to develop new treatment strategies for bladder cancer by regulating miRNA expression levels and copper to zinc levels, which are important markers for prognosis.

There are some limitations of our study. Our findings suggest the miRNA expression profile of a mixed-stage bladder cancer population (ranging from non-muscle invasive bladder cancer to muscle invasive bladder cancer, T1–T4). Consequently, the reported miRNA signature is likely associated with the presence of malignancy itself rather than a specific stage of tumor progression. We cannot definitively assert the utility of the identified miRNA panel for prognosis (i.e., predicting recurrence, progression, or survival), as this requires stratification by stage. The reported findings are strictly limited to the diagnostic value (i.e., distinguishing patients with cancer from healthy controls). The observed expression patterns could obscure stage-specific miRNA changes; therefore, the generalizability of our biomarkers to predict early diagnosis should be interpreted cautiously within the context of symptomatic but un-staged bladder cancer cases. We recognize that future work is essential to dissect the prognostic value of these biomarkers. There should be a sole focus on a homogenous cohort of patients with non-muscle invasive bladder cancer to assess utility in recurrence monitoring. External validation should be conducted using datasets where TNM staging data are comprehensively available to determine stage-specific expression patterns. In addition, we commit to addressing these issues in future studies by focusing on stage-homogenous population and integrating the measurement of key proinflammatory cytokines to better define the independent clinical utility of these miRNA biomarkers.

In our study, important biomarkers for the early diagnosis and treatment of bladder cancer were found. However, there is a need to confirm these findings in different and larger populations and to derive a general profile for the pathogenesis of bladder cancer. Thus, the underlying pathophysiology of bladder cancer will be better understood, and new treatment methods can be developed to prevent bladder cancer.

## 5. Conclusions

Understanding the molecular and epigenetic mechanisms underlying the relationship between inflammatory cytokine signaling and microRNAs in cancer can facilitate the development of new therapeutic strategies targeting the tumor microenvironment. In conclusion, the positive significant correlations between miRNA-21, miRNA-155, and miRNA-34a expression levels in patients with bladder cancer suggest that these miRNAs perform their functions via common or related pathways in cancer pathogenesis. This finding suggests that these miRNAs could be used as common diagnostic or prognostic markers in bladder cancer and could be common therapeutic targets. The positive significant correlations between miRNA-155 and IL-6 and TNF-α might be important in terms of the pathophysiology of disease and potential therapeutic targets. This positive relationship could be explained within the complex network of cancer-associated inflammation. Detailed examination of this significant correlation might contribute to the development of new diagnostic, prognostic, and treatment approaches for bladder cancer. The positive significant correlations between miRNA-34a and IL-6 and TGF-β in patients with bladder cancer could be due to the complexity of the cancer microenvironment, the structure of molecular signaling networks, or the special functions in the binding of miRNAs. Furthermore, there might be a complex relationship between the serum copper to zinc ratio and TGF-β in patients with bladder cancer, and these two factors might interact with each other within the context of inflammation and oxidative stress in the tumor microenvironment. Therefore, the mechanisms underlying these specific correlations in patients with bladder cancer need to be elucidated. The significant negative correlation between the serum copper to zinc ratio and TGF-β in patients with bladder cancer could be explained by the dual role of TGF-β in cancer progression and the dynamic changes in trace elements in the tumor microenvironment. As cancer progresses, a decrease in the tumor suppressive effect of TGF-β or the increase in the serum copper to zinc ratio due to increased angiogenesis and inflammation with the development of resistance might create this negative correlation. This result is important for investigating the serum copper to zinc ratio as a prognostic marker in bladder cancer and the potential of TGF-β-targeted therapies.

## Figures and Tables

**Table 1:** Comparison of clinical and demographic findings between patients diagnosed with bladder cancer and control groups.

Clinical and Demographic Parameters	Patient Group (n = 75)	Control Group (n = 75)	Odds Ratio (95% Confidence Interval)	*p* Value
Hypertension (+)	42 (56.0%)	11 (14.7%)	7.405 (3.376–16.244)	<0.001 ^a^*
Diabetes (+)	21 (28.0%)	12 (16.0%)	2.042 (0.920–4.530)	0.115 ^a^
Cholesterol (+)	14 (18.7%)	3 (4.0%)	5.508 (1.512–20.065)	0.010 ^a^*
Heart disease (+)	24 (32.0%)	3 (4.0%)	11.294 (3.227–39.526)	<0.001 ^a^*
Familial history of cancer (+)	36 (48.0%)	13 (17.3%)	4.402 (2.079–9.321)	<0.001 ^a^*
Familial history of bladder cancer (+)	14 (18.7%)	2 (2.7%)	8.377 (1.832–38.306)	0.004 ^a^*
Alcohol (+)	25 (33.3%)	21 (28.0%)	1.286 (0.641–2.579)	0.595 ^a^
Smoking (+)	44 (58.7%)	21 (28.0%)	3.650 (1.845–7.219)	<0.001 ^a^*

^a^ Chi-squared test, logistic regression. (+): available. *: statistically significant (*p* < 0.05).

**Table 2:** Comparison of miRNA-21, miRNA-155, miRNA-34a, IL-6, TNF-α, TGF-β, iron, copper, zinc, and copper to zinc ratio levels between patients diagnosed with bladder cancer and control groups.

	Patient Group (n = 75)		Control Group (n = 75)		*p* Value
	Mean	Standard Deviation	Mean	Standard Deviation	
Age (years)	66.15	5.93	64.00	6.37	0.034 ^a^*
miRNA-21	16.40	4.76	1.90	1.08	<0.001 ^b^*
miRNA-155	4.06	6.19	1.13	1.05	<0.001 ^b^*
miRNA-34a	1.01	0.90	1.54	0.42	<0.001 ^b^*
IL-6	46.37	18.12	18.58	14.92	<0.001 ^b^*
TNF-α	134.81	39.31	99.99	21.88	<0.001 ^b^*
TGF-β	12.07	5.70	8.19	1.68	<0.001 ^b^*
Iron	96.39	6.61	129.84	5.19	<0.001 ^a^*
Copper	167.41	17.33	132.98	6.30	<0.001 ^a^*
Zinc	99.33	13.44	124.70	5.73	<0.001 ^a^*
Copper to zinc ratio	1.71	0.29	1.07	0.07	<0.001 ^a^*

^a^ Independent samples *T*-test. ^b^ Mann–Whitney U test. *: statistically significant (*p* < 0.05).

**Table 3:** Comparison of miRNA-21, miRNA-155, miRNA-34a, IL-6, TNF-α, TGF-β, iron, copper, zinc, and copper to zinc ratio values among patients diagnosed with bladder cancer.

miRNAs, Proinflammatory Cytokines and Serum Trace Elements		miRNA-21	miRNA-155	miRNA-34a	IL-6	TNF-α	TGF-β	Iron	Copper	Zinc	Copper to Zinc Ratio
miRNA-21	*r*	1.000	0.232 *	0.382 **	0.157	0.200	0.096	−0.167	−0.031	0.128	−0.051
	*p*		0.046	0.001	0.180	0.085	0.410	0.153	0.791	0.274	0.667
miRNA-155	*r*	0.232 *	1.000	0.176	0.279 *	0.325 **	0.146	−0.152	−0.041	−0.015	−0.024
	*p*	0.046		0.132	0.015	0.004	0.213	0.194	0.727	0.897	0.835
miRNA-34a	*r*	0.382 **	0.176	1.000	0.294 *	0.136	0.447 **	−0.142	−0.129	0.178	−0.196
	*p*	0.001	0.132		0.010	0.245	0.000	0.225	0.268	0.128	0.092
IL-6	*r*	0.157	0.279 *	0.294 *	1.000	0.102	0.356 **	0.044	−0.044	−0.145	0.075
	*p*	0.180	0.015	0.010		0.385	0.002	0.708	0.705	0.215	0.522
TNF-α	*r*	0.200	0.325 **	0.136	0.102	1.000	0.121	−0.160	0.035	0.087	−0.004
	*p*	0.085	0.004	0.245	0.385		0.303	0.170	0.767	0.458	0.972
TGF-β	*r*	0.096	0.146	0.447 **	0.356 **	0.121	1.000	0.066	−0.201	0.094	−0.235 *
	*p*	0.410	0.213	0.000	0.002	0.303		0.574	0.084	0.423	0.042
Iron	*r*	−0.167	−0.152	−0.142	0.044	−0.160	0.066	1.000	−0.013	−0.139	0.113
	*p*	0.153	0.194	0.225	0.708	0.170	0.574		0.910	0.234	0.333
Copper	*r*	−0.031	−0.041	−0.129	−0.044	0.035	−0.201	−0.013	1.000	−0.046	0.675 **
	*p*	0.791	0.727	0.268	0.705	0.767	0.084	0.910		0.694	0.000
Zinc	*r*	0.128	−0.015	0.178	−0.145	0.087	0.094	−0.139	−0.046	1.000	−0.669 **
	*p*	0.274	0.897	0.128	0.215	0.458	0.423	0.234	0.694		0.000
Copper/Zinc	*r*	−0.051	−0.024	−0.196	0.075	−0.004	−0.235 *	0.113	0.675 **	−0.669 **	1.000
	*p*	0.667	0.835	0.092	0.522	0.972	0.042	0.333	0.000	0.000	

*: correlation is statistically significant to *p* < 0.05. **: correlation is statistically significant to *p* < 0.01.

## Data Availability

The data presented in this study are available on request from the corresponding author. The data are not publicly available due to ethical reasons.

## Abbreviations

The following abbreviations are used in this manuscript:

RT-PCR	Real-time polymerase chain reaction
EDTA	ethylenediaminetetraacetic acid
